# NeuralBeds: Neural embeddings for efficient DNA data compression and optimized similarity search

**DOI:** 10.1016/j.csbj.2023.12.046

**Published:** 2024-01-15

**Authors:** Oluwafemi A. Sarumi, Maximilian Hahn, Dominik Heider

**Affiliations:** a Department of Mathematics and Computer Science, University of Marburg, Hans-Meerwein-Str. 6, Marburg, D-35043, Germany; b Institute of Computer Science, Heinrich-Heine-University Duesseldorf, Graf-Adolf-Str. 63, Duesseldorf, D-40215, Germany

**Keywords:** DNA similarity, Neural embeddings, Artificial intelligence

## Abstract

The availability of high throughput sequencing tools coupled with the declining costs in the production of DNA sequences has led to the generation of enormous amounts of omics data curated in several databases such as NCBI and EMBL. Identification of similar DNA sequences from these databases is one of the fundamental tasks in bioinformatics. It is essential for discovering homologous sequences in organisms, phylogenetic studies of evolutionary relationships among several biological entities, or detection of pathogens. Improving DNA similarity search is of outmost importance because of the increased complexity of the evergrowing repositories of sequences. Therefore, instead of using the conventional approach of comparing raw sequences, e.g., in fasta format, a numerical representation of the sequences can be used to calculate their similarities and optimize the search process. In this study, we analyzed different approaches for numerical embeddings, including Chaos Game Representation, hashing, and neural networks, and compared them with classical approaches such as principal component analysis. It turned out that neural networks generate embeddings that are able to capture the similarity between DNA sequences as a distance measure and outperform the other approaches on DNA similarity search, significantly.

## Highlights

• CNNs trained with ladder loss provide viable embeddings for efficient DNA similarity search.
• Trained NNs create a well structured vector latent space for DNA similarity search.
• Neural embedding can efficiently capture the information in DNA sequence as vectors and reduce the storage disk space.

## Graphical abstract

**Figure:**
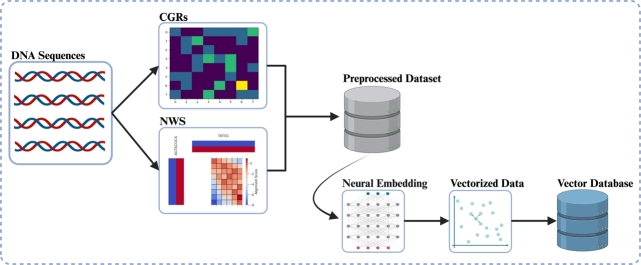

## 1 Introduction

The rapid progress in high-throughput Next Generation Sequencing (NGS) tools and technologies, exemplified by platforms like Illumina or Nanopore, has ushered in an unprecedented era of biological data generation. This surge encompasses diverse types of biological information, including DNA, RNA, and protein sequences. The transformation in the generation and analysis of biological datasets has led to a remarkable proliferation of omics data. These extensive datasets find their curation in various databases, notable among them being the National Center for Biotechnology Information (NCBI), Ensembl, the European Molecular Biology Laboratory (EMBL) sequence repository, and UniProt.

These databases serve as valuable resources for numerous essential bioinformatics tasks, such as DNA similarity search [1], sequence alignments [2], gene annotation [3], [4], gene prediction [5], [6], and motif finding [7], [8]. However, as these databases store vast volumes of sequences, performing these bioinformatics tasks is becoming increasingly challenging and complex. Towards reducing the complexities involved in searching large biological databases for similar DNA sequences and other bioinformatics tasks, various vector embedding techniques can be explored to generate lower dimensional vectors of the sequences for efficient storage and retrieval from a vector database. Vector databases [9], [10] have become more popular among researchers, providing platforms for data points to be embedded in a vector space using embedding functions [11], [12] instead of storing the data in tables using the usual relational databases. Some of the widely used embedding techniques for biomedical applications are Local Sensitive Hashing (LSH [13]), Principal Component Analysis (PCA [14]), Frequency Matrix Chaos Game Representation (FCGR [15]), and Artificial Neural Networks (ANNs [16]).

LSH allows for approximate similarity search, which can be beneficial in scenarios where exact matches are not strictly required [13]. This can speed up the search process, especially in large genomic databases. Also, LSH can significantly reduce the computational complexity of similarity search compared to traditional methods like brute-force pairwise comparison, especially when dealing with large datasets. While LSH provides speed and efficiency, it comes at the cost of approximate matching. The degree of approximation needs to be carefully considered based on the specific requirements of the application.

The PCA can help filter out noise and highlight the most significant patterns in the data [14]. This is particularly useful in DNA sequence analysis, where noise may arise from sequencing errors or variations. Also, PCA is computationally efficient, making it suitable for large datasets. It can handle a large number of DNA sequences and reduce the computational burden of subsequent analyse. Nevertheless, PCA involves projecting data onto a lower-dimensional space, leading to some loss of information. The reduced dimensions may not capture all the intricacies of the original DNA sequences. PCA also assumes linearity in the data, which may not hold for complex relationships in DNA sequences.

FCGR is efficient in reducing the dimensionality of DNA sequences, making it suitable for visualization and clustering analyses by employing fractal representation [15]. It suitable for transforming of complex sequence data into a simpler representation. FCGR is generally robust to variations in DNA sequences. However, FCGR have limitations related to loss of sequence order information and sensitivity to sequence length, and this may limit its ability to capture certain types of sequence relationships.

ANNs can discover non-linear relationships and intricate patterns in DNA sequences, which are difficult to identify using traditional methods. Furthermore, they can also learn relevant features from raw DNA sequences, eliminating the need for manual feature engineering when analyzing large datasets [16]. Additionally, ANNs can efficiently scale to handle datasets with large amounts of sequences with varying lengths, a common occurrence with DNA sequences. Furthermore, end-to-end learning with ANNs allows the model to learn hierarchical representations directly from the raw DNA sequence. This enables them to capture both local and global dependencies among the sequences. However, ANNs, especially when dealing with highly complex data such as DNA sequences, are prone to overfitting, where the model may memorize training examples instead of generalizing well to unseen data. ANNs, particularly deep models, are often considered black-box models, making it challenging to interpret the learned features or understand how the model arrives at a particular prediction. Deep Learning (DL) methods such as Convolutional Neural Networks (CNN) are used as embedding functions to create embeddings where similar data points are placed close to each together in a vector space so that similarity search can be highly optimized. For instance, Bee et al. used neural embeddings for images directly encoded into DNA sequences [17] thereby maintaining similarity of the DNA sequences for similar images but without compression.

In our study, we investigated the potential of deep learning models to create neural embeddings that capture DNA sequence similarity as a distance measure while maintaining a reasonable degree of dimensionality reduction that produces viable embeddings suitable for optimizing DNA similarity search, and provide a proof-of-concept for the detection of antimicrobial resistance (AMR) genes in pathogens.

The rise of AMR poses a significant risk to global health, food security, and societal progress. It is estimated that without action against AMR, annual global deaths could reach 10 million by 2050 [18]. In clinical settings, antimicrobial susceptibility testing (AST) is commonly employed for AMR analysis, but it necessitates specialized facilities and trained technicians, limiting its use to bacteria that can be cultured [19]. Recent research has been exploring the use of computational techniques for AMR prediction, combining genomic sequencing with established databases and phenotypic data on AMR [19], [20].

Our findings show that CNN trained with ladder loss show a great potential in creating semantic neural embeddings for DNA sequences that optimize DNA similarity search. DNA similarity search is pivotal to discovering homologous sequences in organisms and phylogenetics study of evolutionary relationships among several biological entities.

Prior to the use of AI algorithms for DNA similarity search, the Needleman-Wunsch [21] and Smith-Waterman [22] algorithms have been widely used in computing DNA sequence similarity either as a global or local alignment. The Needleman-Wunsch algorithm is designed to find the optimal global alignment between two sequences, while the Smith-Waterman algorithm is used for local alignment, i.e., for finding motifs within the sequences. Although the Needleman-Wunsch and Smith-Waterman generate optimal global and local alignments respectively; nevertheless, the algorithms' running time complexity is the key limitation when dealing with large sequence databases. Heuristic algorithms such as Basic Local Alignment Search Tool (BLAST) [23], [24], FASTA [25], and DIAMOND [26] have been widely embraced for obtaining DNA similarity in a timely way.

BLAST employs a seed-and-extend algorithm, which breaks the query sequence into small segments (seeds) and quickly identifies matches in the database before extending the alignment. FASTA, on the other hand, uses a word search algorithm that compares segments (words) of the sequences in a pairwise manner. Both BLAST and FASTA use scoring matrices to assign scores to matches and mismatches during sequence alignment. However, the specific scoring matrices used can vary. BLAST often utilizes the BLOSUM [27] as the substitution matrix for protein sequences, but users can specify other protein scoring matrices such as PAM [28]. FASTA typically employs the PAM or substitution matrices derived from its own alignments. Both tools provide statistical measures like E-values to estimate the significance of sequence similarities. BLAST is generally faster and more sensitive for large-scale database searches, while FASTA is often favored for custom databases and specific research needs. Despite the potential of BLAST and FASTA algorithms, the low accuracy of their search results due to the heuristic nature of the algorithm is a drawback.

To increase the speed and accuracy of DNA similarity search and other related bioinformatics tasks, ML techniques have been proposed as a sturdy approach for developing viable computational tools. ML techniques [29], especially deep learning [30], have been explored to develop models for DNA sequence analysis and similarity search. This study explored two ANN approaches in creating neural embeddings; the fully connected networks (FCN) [31] and CNN [32] trained with both triplet loss and ladder loss. FCNs and CNNs are two prevalent types of ANN used in deep learning models. While both types of networks can learn and make predictions, they have different architectures.

In an FCN, also known as a dense network or multi-layer perceptron (MLP), each neuron is connected to every neuron in the previous and subsequent layers. This means that the input to each neuron is a weighted sum of the outputs of all neurons in the previous layer, followed by an activation function such that predictions are made based on the high-level features extracted by the previous layers. Also, each parameter (weight) in FCN is unique to a specific connection between two neurons. This means that the number of parameters in an FCN can grow rapidly as the size of the input increases and can be computed using matrix multiplication.

On the other hand, CNNs are specifically designed to process grid-like data such as images. They consist of multiple layers, including convolutional, pooling, and fully connected layers. The convolutional layers apply filters to the input data, extracting features by performing convolutions. The pooling layers reduce the spatial dimensions of the data, reducing the computational complexity leveraging on parameter sharing and local connectivity. Also, in the convolutional layers, a small set of weights (kernel/filter) is shared across all spatial locations of the input. This significantly reduces the number of parameters, making CNNs more efficient for processing grid-like data. Both CNN and FCN architectures require training to optimize their parameters. The FCNs must find the optimal values for their matrices and biases, while the CNN needs to learn the best kernels.

Triplet loss [33] and ladder loss [34] functions have been proposed in previous studies for training neural network architectures. The triplet loss is typically used for data with class labels with the key idea of pulling samples of the same class closer together in latent space while simultaneously pushing away samples of different classes. To accomplish this, data is processed as triplets of samples $\left(x,x^{+},x^{-}\right)$, where *x* is called the anchor, and $x^{+}$ and $x^{-}$ are the positive and negative samples, respectively. *x* and $x^{+}$ are selected to be from the same class, while $x^{-}$ is a sample from a different class. One major limitation of utilizing the triplet loss for semantic embedding is the strict and binary distinction between positive and negative samples. By doing so, not all the information provided by the data is utilized. This becomes evident when considering the triplets (A, A, B) and (A, A, C), where s(A,B)≫s(A,C). A has a very high similarity to itself, so B and C are negative samples. Even though A and B are much more similar than A and C, the triplet loss approach will push the sequences B and C away from A by the same margin. Thus, B and C are valued equally, although pushing the more dissimilar sequence C further away would be more appropriate. Therefore, this effect is reduced when learning on the triplet (A, B, C). Nevertheless, training an ANN does not guarantee finding an optimal solution, making this a difficult scenario.

A technique that addresses this oversight in the triplet loss is ladder loss. To apply the ladder loss, all data samples must first be ranked in order of similarity. Also, the ladder loss involves joint optimization of the reconstruction loss and classification loss. The reconstruction loss helps the network to learn robust features, while the classification loss ensures good performance on labeled data.

## 2 Material and methods

**Fig. 1:**
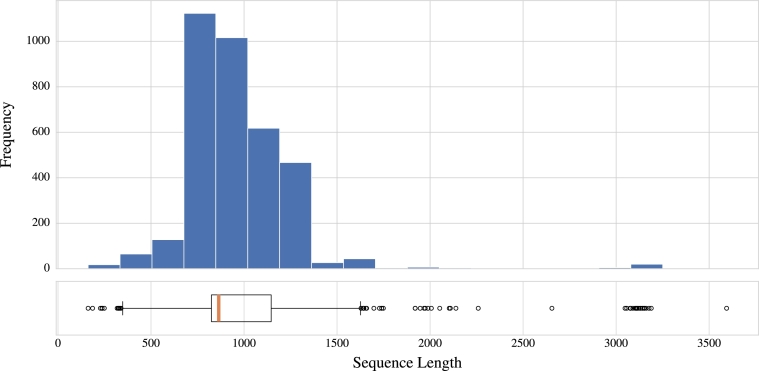
Visualization of the DNA data distribution with sequence lengths spanning from 162 to 3,594 nucleobases. Majority of sequences exhibit similar sizes, clustering around 800 to 1,200 nucleobases, as highlighted in the boxplot diagram.

The dataset used in the study was retrieved from the Comprehensive Antibiotic Resistance Database (CARD) database [35]. Identifying similar DNA sequences from this dataset can benefit Antimicrobial Resistance (AMR) [36] [37] research by providing insights into pathogens' characteristics and their genetic relationship to other pathogens. Also, a quick identification of AMR class for unknown pathogens can drastically improve treatment by providing knowledge about the antibiotics that are likely to be the most effective for the pathogen. The dataset contains 33,860 DNA plasmids from 263 pathogens structured as a collection of sequences and their corresponding GenBank sequence identifier as a label. To remove all potential outliers and noises from the dataset, all pathogens with ten or fewer nucleotide sequences were filtered out. Thus, the cleaned dataset contained 3,549 DNA sequences from 47 distinct pathogens with lengths ranging from 162 to 3,594 nucleotides and most sequences of a similar size, around 800 to 1,200 nucleotides, with only a few exceptionally long sequences, as shown in Fig. 1.

Also, given that the DNA sequences are of varying lengths and comprised of non-digit characters, it is necessary to convert them to a form suitable for machine learning operations. Therefore, we transformed them into a uniform representation using the chaos game representation (CGR) [38] such that all the sequences are represented as CGR images with the same resolution. The main idea of using the CGRs is to map a one-dimensional sequence of categorical values into a two-dimensional polygon, where every vertex represents one of the unique categorical values. First, a regular polygon with as many vertices as there are unique values that compose the sequences is created. Each unique categorical input value is then assigned to a vertex. Starting from the center of the polygon, a marker is placed. For every value in the sequence, the marker is moved halfway between its current position and the vertex corresponding to the sequence's current value. Its position is marked with a dot in the polygon, and the maker's movement creates the CGR images by iterating through the whole sequence. It has been shown recently that CGR-encoding can lead to superior performance in subsequent machine learning [15], [39].

**Fig. 2:**
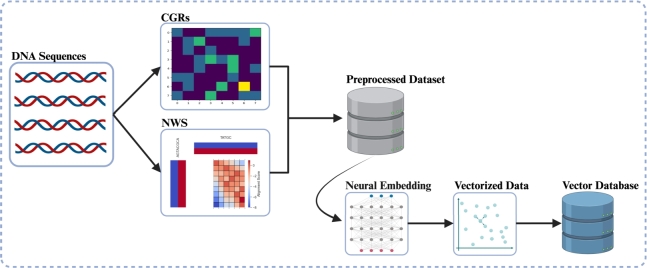
Visualization of the system flowchart encompassing the preprocessing of input DNA sequences and the utilization of ANN to generate neural embeddings stored in the vector database. Figure was created with Biorender.com.

**Fig. 3:**
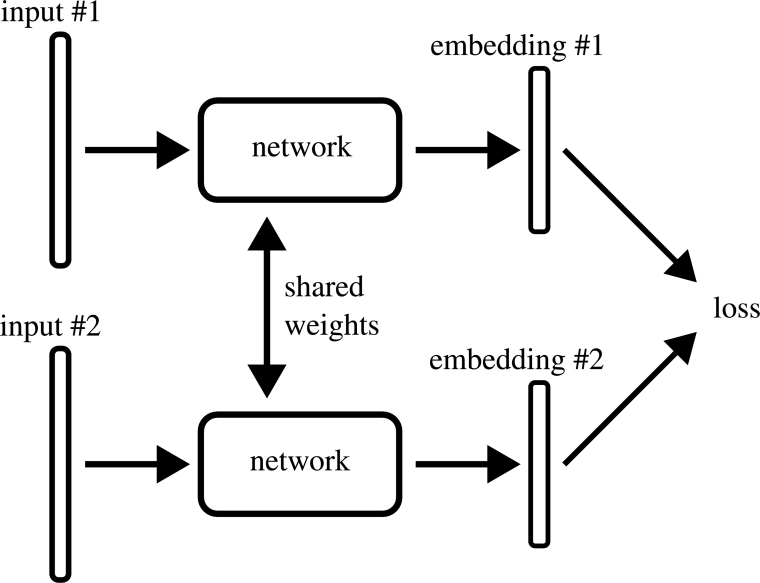
Visualization of the Siamese Neural Network Structure used for training the CNN and FCN with the triplet loss and ladder loss functions.

In Fig. 2, the flowchart illustrates the algorithmic process for generating neural embeddings. To apply the data to similarity search problems effectively, it is essential to determine the ground truth similarity between sequences for accurate ranking. Therefore, we computed the ground truth among sequences utilizing the Needleman-Wunsch (NWS) algorithm with the scoring values of +1 for a match, 0 for mismatches and −1 for gaps (either gap opening or extension). Then, the obtained NWS data and CGRs were combined to generate the input dataset for the ANN; such that each entry has the structure $\left(q,(a_{1},s_{1}),...,(a_{n},s_{n})\right)$. Where, *q* refers to the anchor sequence, whose embedding will be compared to the embeddings of sequences $a_{1}$ to $a_{n}$. Also, $s_{i}:=s(q,a_{i})$ represent the computed NWS, where $s_{i}\geq s_{j}$ for i<j. Hence, the order of the comparison sequences $a_{1}$ to $a_{n}$ is equivalent to the ranking by highest similarity. Since the dataset has 3,549 entries, each of the original DNA sequences is used as an anchor sequence exactly once. Also, since neural embeddings need training data to adjust the ANN's weights, we obtained 80% of all the data for training and 20% for testing. The train-test split is based only on the anchor sequence *q* and does not consider the comparison sequences $a_{1},...,a_{n}$. We explored two ANN techniques (FCN and CNN) with two loss functions (triplet loss, and ladder loss) to create vector representations of DNA sequences using a Siamese Neural Network (SNN) [40] architecture as shown in Fig. 3. SNNs are designed for binary classification tasks in which input data comes in pairs and the goal is to determine similarity or dissimilarity. Usually they consist of two mirrored sub-networks sharing weights, but can alternatively feature several sub-networks. Our approach involves two sub-networks consisting of CNNs or FCNs. Each sub-network is presented with a different input and then the outputs are processed with a loss function (triplet loss or ladder loss), adjusting the weights so the SNN minimizes the loss. This produces embeddings that maintain similarity in latent space. Then the models are trained by running input data through the network, calculating a loss based on similarity in latent space, and backpropagating the error through the network. This pulls similar sequences together and pushes dissimilar sequences apart in the latent space.

### 2.1 Embedding techniques

#### 2.1.1 Triplet loss function with FCN and CNN

The application of FCN and CNN with triplet loss function required training of the input sequence. Table 1 provides details on the architectural layouts and optimal hyperparameter settings for training both the CNN and FCN. Also, dropout layers serves as a regularization technique incorporated into the models to mitigate the risk of overfitting during training. Additionally, early stopping is employed, which intervenes in the training process if the validation loss begins to rise, signaling potential overfitting of the model to the training data. However, models using the triplet loss cannot be trained directly on samples in the form of $\left(q,(a_{1},s_{1}),...,(a_{n},s_{n})\right)$. The training of the two ANN models requires triplets of the form $\left(x,x^{+},x^{-}\right)$, where *x* is the anchor sequence, $x^{+}$ is a positive sample, and $x^{-}$ is a negative sample. Therefore, for each anchor sequence *q*, the corresponding comparison sequences are used to create a set of triplets $T_{q}=U_{i=1}^{y}U_{j=i+1}^{n}(q,a_{i}^{+},a_{j}^{-})$. This set encompasses all possible triplets for anchor *q*, where the similarity of the positive sample is higher than that of the negative sample, such that the dataset of the triplets for all anchors $T_{q}=U_{q}T_{q}$ was employed to train the triplet loss models. Let *T* be the dataset of all possible triplets, with a cardinality of *N*. The loss *L* aims to minimize the distance from the anchor to the positive sample and to maximize the distance to the negative sample for all triplets in *T* as shown in Eq. (1).

(1) $$L=\sum_{i}^{N}{\left[||f(x_{i})-f({x_{i}}^{+})|{|}_{2}^{2}-||f(x_{i})-f({x_{i}}^{-})|{|}_{2}^{2}+\alpha \right]}_{+}$$

Where *f* stands for the embedding function, represented by the network, and *α* is a margin that defines how significant the distance between positive and negative samples should be. Also, a closer look at Eq. (1) makes it apparent that the loss is minimized when the distance between the anchor and positive sample is small and between the anchor and negative sample is high. Using the non-negative portion of each summand ensures that the loss cannot be improved by pushing the negative sample infinitely far away.

**Table 1:** Optimal-performing CNN and FCN Architectures layout along with their corresponding hyperparameter configurations.

Layers	CNN	FCN
Convolutional	Neurons: 1024	Not Applicable
	Kernel Size: 5	
	Activation Function: ReLU	

Pooling	Padding: 0	Not Applicable
	Strides: 1	

Flattening	None	Not Applicable

Fully Connected	Neurons:512	Neurons: 512
	Activation Function : ReLU	Activation Function: ReLU
	Dropout: 0.2	Dropout:0.2
	Kernel size: 3	Kernel size: 3

Output	Activation Function: tanh	Activation Function : ReLU
	Learning rate: 6e-7	Learning rate: 6e-7

#### 2.1.2 Ladder loss function with FCN and CNN

A train-test split dataset is also required for utilizing the ladder loss function in FCN and CNN architectures. However, while the triplet loss requires triplets $\left(x,x^{+},x^{-}\right)$ to train, the ladder loss requires groups of similar sequences $N_{i}^{-q}$ ranked in order of similarity. Given an anchor *q*, the remaining data can be divided into *M* groups $N_{1}^{-q},...,N_{M}^{-q}$, where the samples in $N_{i}^{-q}$ are more similar to *q* than samples in $N_{j}^{-q}$ for i<j such that $N_{a:b}^{-q}$ := $U_{i=a}^{-q}$ $N_{i}^{-q}$, 1 ≤a,b≤M∈N. The loss in each group $L_{lad}^{i}$ can be calculated as a triplet loss where the group's samples are considered positive, samples of more dissimilar groups are considered negative, and *q* remains the anchor which represents a general case as shown in Eq. (2)

(2) $$L_{lad}^{i}(q)=\sum_{x^{+}\in N_{i-1}^{-q}}\sum_{x^{-}\in N_{i:M}^{-q}}{\left[\alpha_{i}-s(q,x^{+})+s(q,x^{-})\right]}_{+},i\in \{2,...,M\}$$

In the second scenario of $L_{lad}^{1}$, all the samples aside from *q* are considered as negative; representing a special case since the only positive sample is *q* itself, and thus, all other samples are pushed away equally as shown in Eq. (3)

(3) $$L_{lad}^{1}(q)=\sum_{x^{-}\in N_{i:M}^{-q}}{\left[\alpha_{1}-s(q,x^{+})+s(q,x^{-})\right]}_{+}$$

After calculating the losses for each group, they can be used to create the final loss, which is used to measure the model's error. As shown in Eq. (4), the final ladder loss for a given anchor *q* is the weighted sum of each group's loss. The weights $\beta_{1},...,\beta_{M}$, as well as the margins $\alpha_{1},...,\alpha_{M}$ are optimizable hyperparameters that influence the importance of correct ordering between different groups and determine how far the groups are pushed apart. Also, ladder loss allows the data points to freely organize themselves by not grouping them with other data points and offers a flexible approach to ranking the DNA sequences.

(4) $$L_{lad}(q)=\sum_{i=1}^{M}\beta_{i}L_{lad}^{i}(q)$$

### 2.2 Evaluation metrics

In this study, one of our goals is to determine the veracity of neural embeddings in extracting and compressing the necessary similarity information contained within the CGR image to capture DNA sequence similarity in a latent space. Therefore, the embedding methods were evaluated based on the information retrieval task by employing the generated embeddings of each model to calculate their similarity in latent space. Given the ground truth sequence similarity and the sequences' calculated similarities, the sequences' actual ranking can be compared to the predicted ranking. The predicted ranking of these sequences is obtained by calculating the similarity between embeddings and sorting the data based on this measurement. Since all the embeddings are vector representations of the sequences, the cosine similarity was used to obtain the similarity score of the vectors as shown in Eq. (5).

(5) $$s_{cos}(\overset{\to }{a},\overset{\to }{b}):=\cos\theta =\frac{\overset{\to }{a}.\overset{\to }{b}}{\left||\overset{\to }{a}||||\overset{\to }{b}|\right|}$$

Also, the quality of the embedding techniques can be measured by quantifying their ranking performance. Therefore, we utilized the Normalized Discounted Cumulative Gain (NDCG), based on the Discounted Cumulative Gain (DCG) as shown in Eq. (6), to obtain the ranking performance of the embedding techniques. The NDCG calculates a ranking score in a way that gives more importance to correct predictions on higher ranks rather than valuing each rank the same.

(6) $$DCG=\sum_{i=1}^{\left|R\right|}\frac{rel_{i}}{log(i+1)}$$

where *R* is the set of embedded sequences ordered by predicted similarity, and $rel_{i}$ represent the importance of the embedding at position iinR. In this study, $rel_{i}$ was calculated as the reversed rank of the embedding: n−i+1. However, the NDCG does not give a direct indication of how good the ranking is for the most similar sequences, which is a vital parameter when considering semantic embeddings. Therefore, we calculated the positional rank score as shown in Eq. (7) for predicting a position *p* similar to the mean squared error but punishes the errors more severely due to the exponential term.

(7) $$score_{p}=\frac{1}{N}\sum_{i=1}^{N}\frac{1}{e^{\left|p-true_{p}^{i}\right|}}$$

Where *N* is the number of entries in the dataset, and $p-true_{p}^{i}$ is the true rank of the CGR at position *p* of the i−th data entry. Therefore, this score takes into account the prediction for rank *p* of every dataset entry and measures how far they deviate from their true rank.

## 3 Results

We evaluated the performance of our method by comparing its results to three embedding techniques: FGCR [15], PCA [14], and LSH [13] using three variants [41]: LSH with average hashing (ahash), LSH with perceptual hashing (phash), and LSH with wavelet hashing (whash). The same dataset and computational setup were used for the comparison. The models were compared based on the quality of the embeddings they generated, which stems from their ability to encode similarity among sequences. Secondly, we assessed the degree to which the models can compress the DNA sequence without losing sensitive information. Thirdly, we analyzed the time the models took to generate valuable embeddings. The FCGR was used as the baseline model for comparing the performances of other models.

**Fig. 4:**
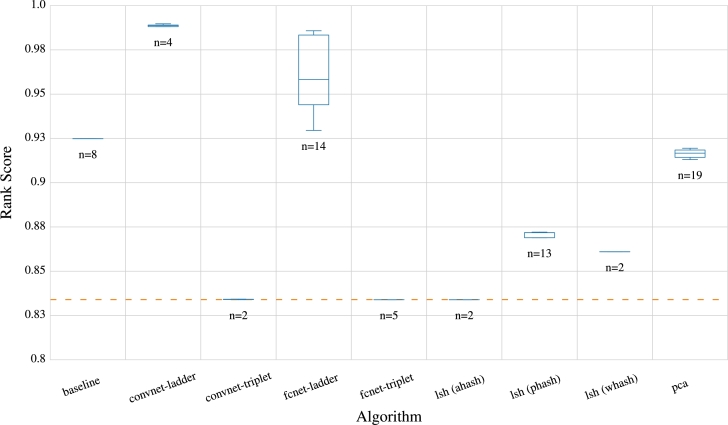
Visualization of NDCG rank scores for different models reveals that, among the embedding approaches, FCGR serves as the baseline model and outperforms other methods, except the neural embeddings. The neural embeddings using ladder loss (convnet-ladder) exhibit the highest overall score, significantly surpassing the baseline and all other models. n represents the number of runs. For some approaches, n is smaller, e.g., the hashes, because there is no variance in the results compared to the deep learning architectures.

**Fig. 5:**
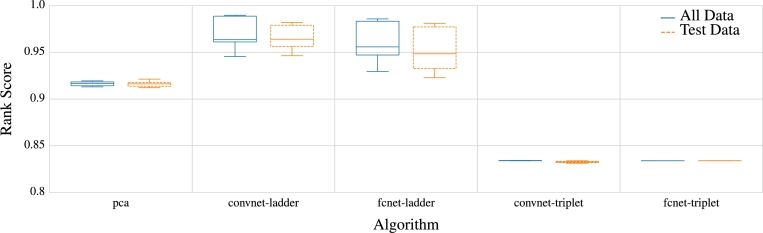
Visualization of the generalization capacity of models across the entire dataset indicates that models trained with triplet loss, including both CNN and FCN, exhibit poor performance. On the contrary, models trained with ladder loss, both CNN and FCN, showcase the highest generalization ability.

In the assessment of the generated embeddings' qualities, Fig. 4 illustrates the evaluation outcomes of the models based on the NDCG score, computed from the evaluation data derived from the source dataset. Notably, the baseline model outperforms the majority of other approaches, with only neural embeddings using ladder loss achieving higher average scores and exhibiting a significant lead in overall performance. Also, to generalize our models, we compared the results of NDCG ranking over the entire data set with the train-test split method. It is evident from the results in Fig. 5 that the triplet-loss models that performed poorly over the whole dataset. As for the results of PCA, they do not deviate notably from one another, although the performance on the test set exhibits slightly higher variance. In the case of the ladder loss function, they continue to demonstrate good results, even on the test set, although the ranking performance on the test set is slightly lower than the overall performance. Notwithstanding, the results of the CNNs are more stable and closer to the overall dataset's scores than the FCN model. Consequently, the ladder loss CNNs model gives the best output and can be regarded as a generalization model for the entire data and the train-split dataset. However, for the approaches that utilize the train-test split, the performance on the test set becomes a crucial indicator of the technique's generalizability.

**Fig. 6:**
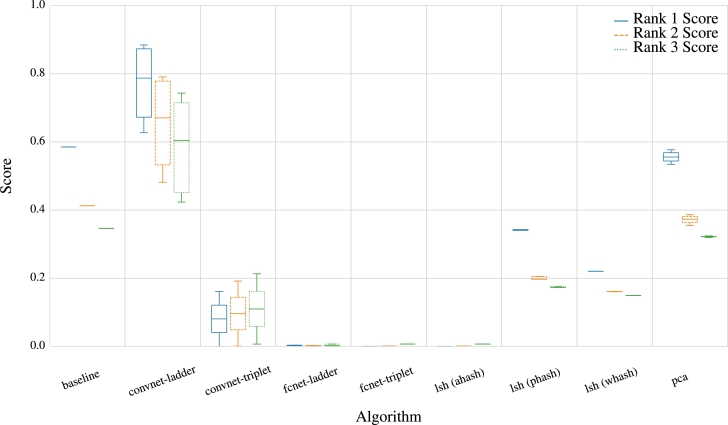
Visualization of positional rank scores for ranks one (left bar), two (center bar), and three (right bar) reveals that CNNs with ladder loss consistently achieve the highest scores. Notably, this technique is the only one that outperforms the baseline model.

Moreover, in Fig. 6, instead of the NDCG rank score, we utilized positional rank scores for ranks one (left bar), two (center bar), and three (right bar). The outcomes reveal that CNN trained with ladder loss achieved the highest scores, emerging as the sole model surpassing the baseline model's performance. While these overall trends align with our observations in Fig. 4, there is substantial variation in the performance of FCN trained with ladder loss. Despite FCN's ladder loss showing commendable results with the NDCG score, as depicted in Fig. 4, the positional rank scores exhibit considerable variance and an overall subpar performance. Thus, while FCN with ladder loss may exhibit reasonably good overall rankings, it proves to be one of the less effective approaches in accurately identifying the exact rank of a sequence. Additionally, Fig. 6 illustrates that, for most approaches, the prediction of rank one surpasses the prediction of rank two, and both outperform predictions for rank three. This pattern is disrupted only by the least performing approaches, particularly evident for CNNs trained with triplet loss.

**Fig. 7:**
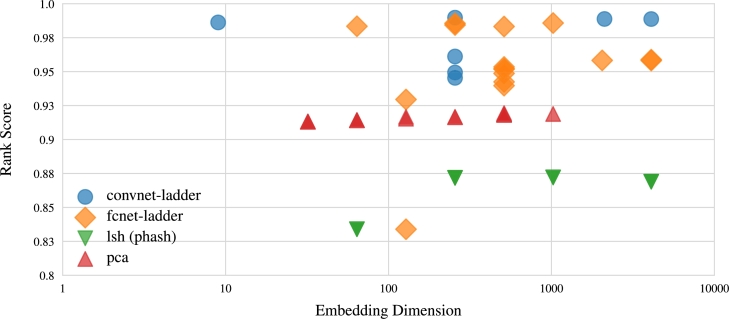
Visualization illustrating the NDCG scores of the model's performance in relation to embedding dimensions for compressed DNA sequences. The results highlight the superior capacity of neural embeddings to efficiently embed sequences into lower-dimensional latent spaces.

**Fig. 8:**
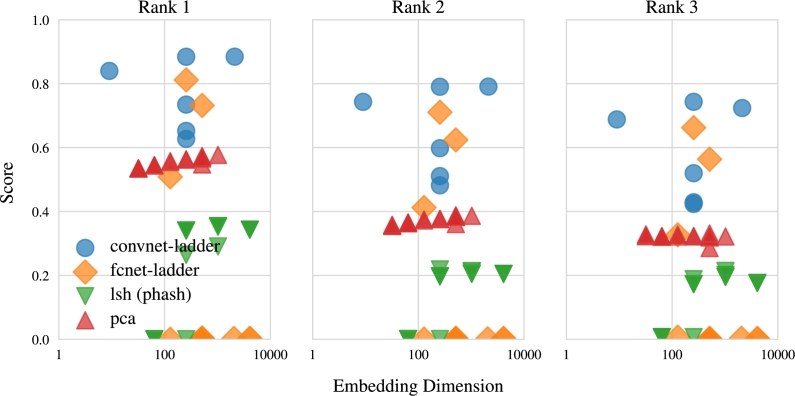
Visualization of the positional rank scores of the model's performance relating to embedding dimensions for compressed DNA sequences. The results indicate that larger embedding sizes generally result in higher rank scores, with the neural embedding using ladder loss (convnet-ladder) showcasing superior performance across all three positions.

Secondly, when assessing the models' ability to compress DNA sequences, the rank scores for various embedding dimensions in each model, depicted in Fig. 7, Fig. 8, indicate that smaller embedding dimensions result in lower rank scores. For example, both PCA and CNNs exhibit a gradual decline in scores as sequences are embedded into fewer dimensions. While FCN's rank scores do not strictly conform to this pattern, the overall trend aligns with the concept. LSH also conforms to the trend of diminished performance with lower-dimensional embeddings, akin to random guessing when reducing data to 64 dimensions. Moreover, Fig. 7 underscores that neural embeddings excel in embedding sequences into lower-dimensional latent spaces. In essence, the patterns observed in Fig. 7, Fig. 8 underscore the necessity of finding a balance between substantial data compression and retaining essential information.

**Fig. 9:**
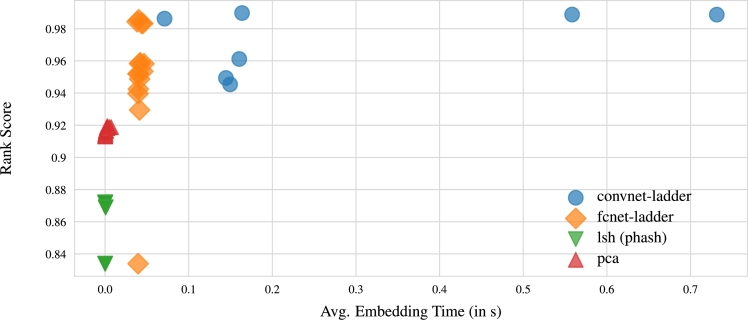
Visualization illustrating the rank scores relative to the average time taken to create sequence embeddings. This result shows that PCA and LSH emerge as the fastest methods for generating sequence embeddings. However, these methods produce lower-quality embeddings. In contrast, convnet-ladder generates superior quality embeddings but requires a longer processing time.

**Fig. 10:**
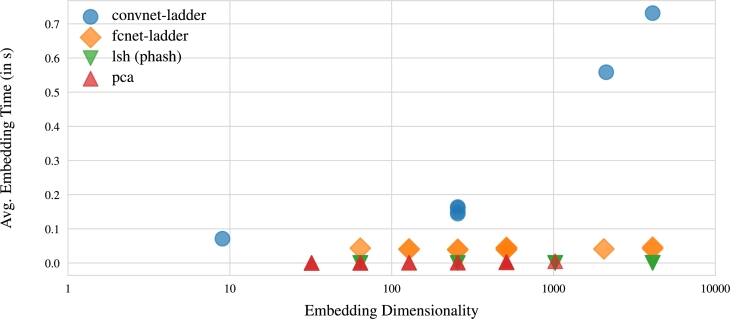
Visualization depicting the relationship between embedding time and embedding dimensions. The outcomes indicate that PCA, LSH, and fcnet-ladder maintain a consistent average embedding time across various embedding sizes. Conversely, the convnet-ladder exhibits a gradual increase in processing time as the embedding size grows.

Thirdly, another crucial aspect used to assess model performance is the time taken to embed a given DNA sequence. In Fig. 9, where the rank score was plotted against the embedding time, the outcomes reveal that PCA and LSH stand out as the fastest techniques for embedding CGR images of DNA sequences. However, it's noteworthy that they produce low-quality embeddings, as illustrated in Fig. 4; this aligns with expectations since they are lightweight, unparameterized approaches. In contrast, the CNN model requires the longest time to embed a CGR image of the sequence. Unlike other techniques, which exhibit slight variance in their data embedding time, CNN displays significant variability. Nevertheless, the results presented in Fig. 10, where embedding time was compared with embedding dimension, indicate that other models are not affected by the embedding size. In contrast, CNNs become progressively faster at embedding as the dimensionality decreases. Therefore, it's clear that for CNNs, the time needed to embed data is influenced by the embedding dimension.

**Fig. 11:**
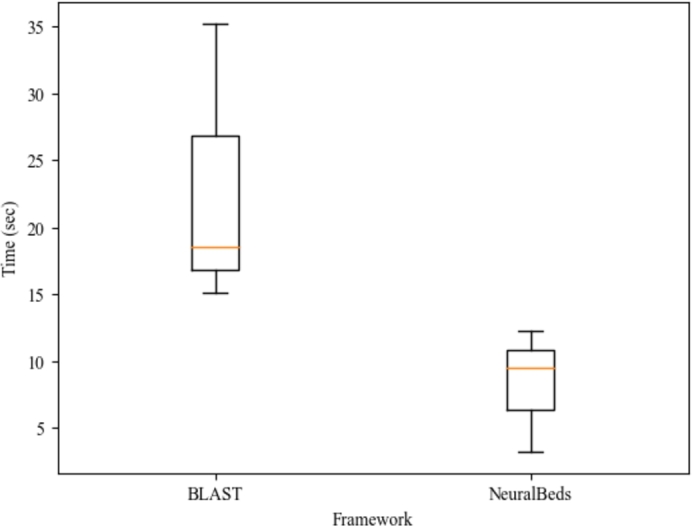
Visualization of the retrieval time of NeuralBeds compared to BLAST demonstrates that NeuralBeds returns similar DNA sequences within a shorter time frame than BLAST.

As a use case, we compared the performance of our CNN neural embedding with ladder loss (NeuralBeds) to BLAST in terms of retrieval speed, disk storage usage and quality of the retrieved sequences. Fig. 11 illustrates that NeuralBeds achieved a shorter retrieval time than BLAST. Moreover, NeuralBeds demonstrates an increased sensitivity of 89%, surpassing the 74% sensitivity observed in BLAST. Here, sensitivity describes an algorithm's proficiency in capturing all pertinent sequences, thereby minimizing false negatives. This metric is calculated as the ratio of true positives to the sum of true positives (TPs) and false negatives (FNs). Within the framework of BLAST, TPs denote outcomes accurately identified as similar to the query sequence. Hence, it becomes imperative to employ a metric of similarity for the accurate determination of TPs. The determination of TPs relied on the normalized raw score alignment presented in the BLAST report. This score, expressed in bits, is derived by considering both the length and quality of the alignment. Higher scores generally indicate a more robust and reliable similarity between the query and the database sequence. Furthermore, while the BLAST database consumed 15 GB of disk space, the NeuralBeds database required only 1.8 GB for the trained embedding. The comparison utilized 1,265,047 FASTA DNA sequences of salmonella bacteria downloaded from the NCBI data repository.

## 4 Discussion

In this study, we delved into the potential of neural embeddings as a viable strategy for optimizing DNA similarity search and archiving. Specifically, we harnessed the power of two neural networks techniques—Convolutional Neural Network (CNN) and Fully Connected Network (FCN)—trained with triplet loss and ladder loss to construct an encoded DNA sequence vector database, thereby enhancing the efficiency of DNA similarity search. The study was driven by key research questions that served as parameters for assessing the feasibility of our methods and interpreting our research findings. These questions revolved around the effectiveness of neural embeddings in capturing DNA sequence similarity, the optimal degree of dimensionality reduction for producing high-quality embeddings, and the comparative speed of various methods in generating DNA embeddings.

Additionally, we conducted a comprehensive comparison of the results obtained from the neural embedding approach with three alternative techniques: Locality-Sensitive Hashing (LSH), Principal Component Analysis (PCA), and Chaos Game Representation (CGR). The evaluation involved a ranking task, where DNA sequences were ordered based on their distance from a query DNA sequence. After examining the results, a number of significant findings have come to light. It is apparent that neural embeddings trained using the ladder loss exhibit superior suitability for semantically embedding DNA sequences. As evidenced by the ranking score, they surpass all other approaches. Notably, this technique stands out as the only one that outperforms unprocessed Chaos Game Representations (CGRs). Furthermore, it demonstrates effectiveness in transforming CGR images across various embedding sizes. However, it is essential to note that the slow embedding time poses a noteworthy drawback, with the model architecture exerting a strong influence on this aspect.

On the contrary, unparameterized techniques like PCA and LSH exhibit speed but fall short in creating sufficiently well-structured latent space embeddings, particularly at higher dimensions. This limitation renders them inadequate for effectively capturing the similarity of DNA sequences. Our observations align with the anticipated outcomes for PCA and LSH, given their unparameterized nature, which does not leverage known similarity information. Consequently, PCA struggles to systematically outperform unprocessed Chaos Game Representations (CGRs) because its transformation matrix extraction disregards real DNA similarities, lacking the incorporation of additional information. Similarly, LSH, being a data-independent approach, does not adapt based on provided DNA similarities. In the realm of neural embeddings, their training specifically targets the real Needleman-Wunsch Score (NWS) between sequences, contributing to their superior performance. Interestingly, models trained with the triplet loss exhibit poor performance, ranking as the least effective among all examined methods. Despite being trained on the similarity relationships of DNA sequences, triplet loss models fail to adequately capture their similarity. In contrast, the findings for ladder loss align with the flexibility and adaptability inherent in ANN for creating neural embeddings. Their capacity to generate well-structured embedding spaces underpins the emergence of vector databases.

Additionally, our observations underscore the influence of embedding dimensions on model performance. Larger embedding dimensions tend to excel in capturing intricate patterns and nuances within DNA sequences, resulting in enhanced overall performance. Conversely, smaller embedding dimensions contribute to information loss and lower rank scores. The augmentation of embedding dimensions equips the model with increased capacity to represent underlying features and relationships in the data. This expanded capacity enables the model to discern finer-grained distinctions between items, consequently improving the accuracy of rankings. With larger embedding dimensions, the model gains the ability to learn more comprehensive representations that encompass a broader range of characteristics. On the flip side, smaller embedding dimensions constrain the expressive power of the model, making it more challenging for the model to capture intricate relationships and patterns within the data. This limitation manifests in lower performance, as the model may struggle to accurately differentiate between items. It's crucial to note that the optimal embedding dimension may vary based on the specific dataset and task at hand. While larger dimensions often yield superior results, there are instances where a smaller dimension suffices or is even preferable, considering constraints such as computational resources or the simplicity of the data.

Furthermore, when considering the speed of generating embeddings, a recurring trade-off between speed and quality becomes apparent, particularly with respect to different models. Neural embedding methods, known for their computational intensity, demand substantial resources for both training and generating embeddings. Consequently, there is an ongoing endeavor to develop expedited methods that can efficiently produce embeddings. These accelerated methods may incorporate techniques like approximation algorithms to hasten the embedding generation process. However, it's important to acknowledge that these speed optimizations sometimes come at the expense of embedding quality. For instance, faster methods might compromise the complexity or depth of the model, resulting in less expressive embeddings. This compromise can lead to a loss of crucial information and details in the data, ultimately affecting the quality of downstream tasks such as ranking. It's crucial to recognize that the specific trade-off between speed and quality can vary based on the task and the specific requirements of the application. In certain scenarios, sacrificing a slight amount of quality for substantial gains in speed may be acceptable or even desirable. Conversely, for tasks where accuracy and quality hold utmost importance—such as DNA similarity search—it becomes imperative to prioritize slower but more accurate embedding methods like neural embedding over unparameterized methods like PCA and LSH.

## 5 Conclusion

This study establishes the feasibility of generating semantic embeddings for DNA sequences in latent space. We explore diverse embedding techniques, investigating their behavior concerning changes in embedding size and the time investment required for their creation. Our findings highlight that the quality of embeddings, relative to their size, is significantly influenced by the chosen technique, typically involving a trade-off between data compression and information retention. Moreover, the time efficiency of embedding creation varies based on the method chosen. Unparameterized approaches like PCA and LSH tend to be faster than neural embedding methods, such as CNN, but they yield lower-quality embeddings, emphasizing the common trade-off between speed and quality. A comprehensive analysis of the results underscores the promise of neural embeddings, particularly those generated by a CNN trained with ladder loss, for embedding DNA sequences in similarity searches. Additionally, we observe that the search runtime for vector representation with neural embeddings is linear, while the runtime for the Needleman-Wunsch algorithm is cubic. This significant efficiency gained from utilizing vector representations instead of traditional Needleman-Wunsch calculations is noteworthy. Once a suitable model is trained, the potential time savings can markedly enhance the efficiency of the search task. To the best our knowledge, this study represents the first exploration of the potential of a CNN trained with ladder loss for DNA data compression and similarity search. Future research can explore new techniques and algorithms to achieve a balance between retrieval time, storage efficiency, and sequence similarity accuracy. Additionally, integration with other bioinformatics tools and databases can expand the capabilities and usefulness of these similarity search tools.

## Declaration of Competing Interest

The authors declare no conflict of interest.
